# Localization of Melatonin Receptor 1 in Mouse Retina and Its Role in the Circadian Regulation of the Electroretinogram and Dopamine Levels

**DOI:** 10.1371/journal.pone.0024483

**Published:** 2011-09-07

**Authors:** Anamika Sengupta, Kenkichi Baba, Francesca Mazzoni, Nikita V. Pozdeyev, Enrica Strettoi, P. Michael Iuvone, Gianluca Tosini

**Affiliations:** 1 Circadian Rhythms and Sleep Disorders Program, Neuroscience Institute and Department of Pharmacology and Toxicology, Morehouse School of Medicine, Atlanta, Georgia, United States of America; 2 Departments of Ophthalmology and Pharmacology, Emory University School of Medicine, Atlanta, Georgia, United States of America; 3 Istituto di Neuroscience, Consiglio Nazionale delle Ricerche, Pisa, Italy; Vanderbilt University, United States of America

## Abstract

Melatonin modulates many important functions within the eye by interacting with a family of G-protein-coupled receptors that are negatively coupled with adenylate cyclase. In the mouse, Melatonin Receptors type 1 (MT_1_) mRNAs have been localized to photoreceptors, inner retinal neurons, and ganglion cells, thus suggesting that MT_1_ receptors may play an important role in retinal physiology. Indeed, we have recently reported that absence of the MT_1_ receptors has a dramatic effect on the regulation of the daily rhythm in visual processing, and on retinal cell viability during aging. We have also shown that removal of MT_1_ receptors leads to a small (3–4 mmHg) increase in the level of the intraocular pressure during the night and to a significant loss (25–30%) in the number of cells within the retinal ganglion cell layer during aging. In the present study we investigated the cellular distribution in the C3H/f^+/+^ mouse retina of MT_1_ receptors using a newly developed MT_1_ receptor antibody, and then we determined the role that MT_1_ signaling plays in the circadian regulation of the mouse electroretinogram, and in the retinal dopaminergic system. Our data indicate that MT_1_ receptor immunoreactivity is present in many retinal cell types, and in particular, on rod and cone photoreceptors and on intrinsically photosensitive ganglion cells (*ip*RGCs). MT_1_ signaling is necessary for the circadian rhythm in the photopic ERG, but not for the circadian rhythm in the retinal dopaminergic system. Finally our data suggest that the circadian regulation of dopamine turnover does not drive the photopic ERG rhythm.

## Introduction

Previous studies have shown that melatonin is synthesized in the eyes of most vertebrates, where it is believed to modulate many important functions [1]. Melatonin exerts its influence by interacting with a family of G-protein-coupled receptors that are negatively coupled with adenylate cyclase [2]. In humans, immunoreactivity to melatonin receptor type 1 (MT_1_) has been located at the photoreceptors, in the inner retinal neurons and on ganglion cells (GCs) [3]. In the mouse, MT_1_ mRNAs have been localized to photoreceptors, inner retinal neurons, and GCs [4]. The distribution of expression suggests that MT_1_ receptors may play an important role in retinal physiology. However, since the vast majority of mouse strains are genetically incapable of synthesizing melatonin in the pineal and retina [5], [6], the effects of melatonin receptor removal on retinal physiology in melatonin-proficient and melatonin-deficient mice are not well understood. Our laboratory has recently produced mice with a targeted deletion of the MT_1_ receptor gene in a melatonin proficient background (C3H/f^+/+^) [4] and we have reported that absence of the MT_1_ receptors has a dramatic effect on the regulation of the daily rhythm in visual processing, and on retinal cell viability during aging [4]. Furthermore, we have also shown that absence of MT_1_ receptors leads to a small (3–4 mmHg) increase in the level of intraocular pressure during the night, and to a significant loss (25–30%) in the number of cells within the retinal GC layer during aging [7].

Previous studies have reported that in C57/Bl6 mice, only the photopic electroretinogram (ERG) is under circadian control, whereas the scotopic ERG is not regulated by the circadian clock [8], [9]. However, since these experiments were performed in melatonin-deficient mice (C57/Bl6), it possible that the lack of circadian regulation in the scotopic ERG may be due to the lack of melatonin signaling. Earlier work has demonstrated that melatonin modulates retinal dopamine release [10], and retinal dopamine content and metabolism are circadian in mice that rhythmically synthesize melatonin, including C3H/f^+/+^ mice, but not in mice that are genetically incapable of synthesizing melatonin [6], [11]–[12]. In melatonin-deficient mice, a circadian rhythm of dopamine metabolism can be induced by daily injections of melatonin [12], and recent findings suggest that melatonin in the mouse retina may contribute to circadian rhythms of protein phosphorylation in photoreceptors [13].

The aim of the present study was to further investigate the cellular distribution of MT_1_ receptors using a newly-developed MT_1_ receptor antibody, and then to determine the role that MT_1_ signaling plays in the circadian regulation of the mouse ERGs and in the retinal dopaminergic system. Our data indicate that MT_1_ receptor immunoreactivity is present in many retinal cell types, and MT_1_ signaling is necessary for the circadian rhythm in the photopic ERG, but not for the circadian rhythm in the retinal dopaminergic system.

## Results

### MT_1_ immunoreactivity in the mouse retina

**Figure 1:**
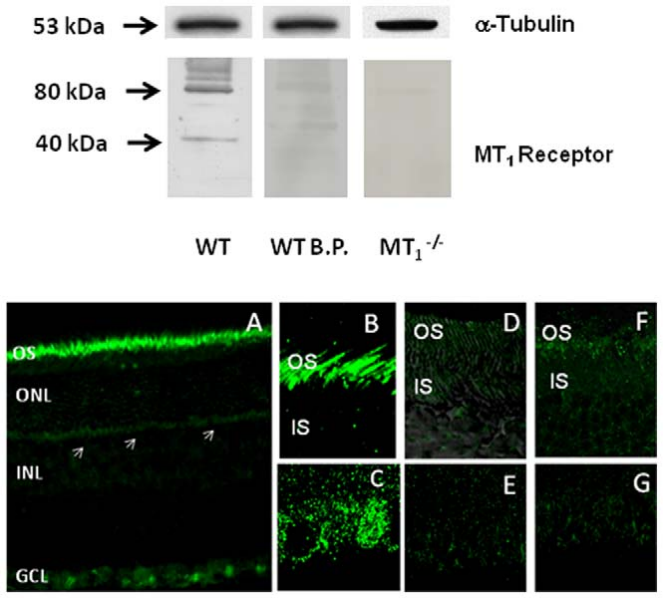
MT_1_ receptor immunoreactivity in the mouse retina. A band corresponding to the molecular weight of approximately 40 kDa was observed in western blotting of retinal extract from WT mice. No band was observed when the blot was treated with antibody pre-absorbed with the blocking peptide. A band of approximately 80 kDa was also present in retinal extract from WT mice. A very faint band around 80 kDa was also present the blot treated with the blocking peptide and in the blot of retinal extract from MT_1_^−/−^ mice. α-Tubulin expression at the molecular weight of 53 kDa is also shown. Ab = Melatonin receptor 1 antibody; BP = blocking peptide. Similar results have been obtained for at least 3 independent samples for each experimental condition (Top panel). MT_1_ immunoreactivity was localized in the outer segments of the photoreceptors (A, B), and in the ganglion cells (A, C), while a weak signal is observed in the outer plexiform layer (A, white arrows). No signal was detected in the outer segments or in ganglion cells using the blocking peptide (D, E) or in retina obtained from MT_1_^−/−^ mice (F, G). OS = outer segments; IS = inner segments; ONL = outer nuclear layer; INL = inner nuclear layer; GCL  = ganglion cell layer. Micrographs are representative of results obtained from at least four animals for each experimental condition (Bottom panel).

**Figure 2:**
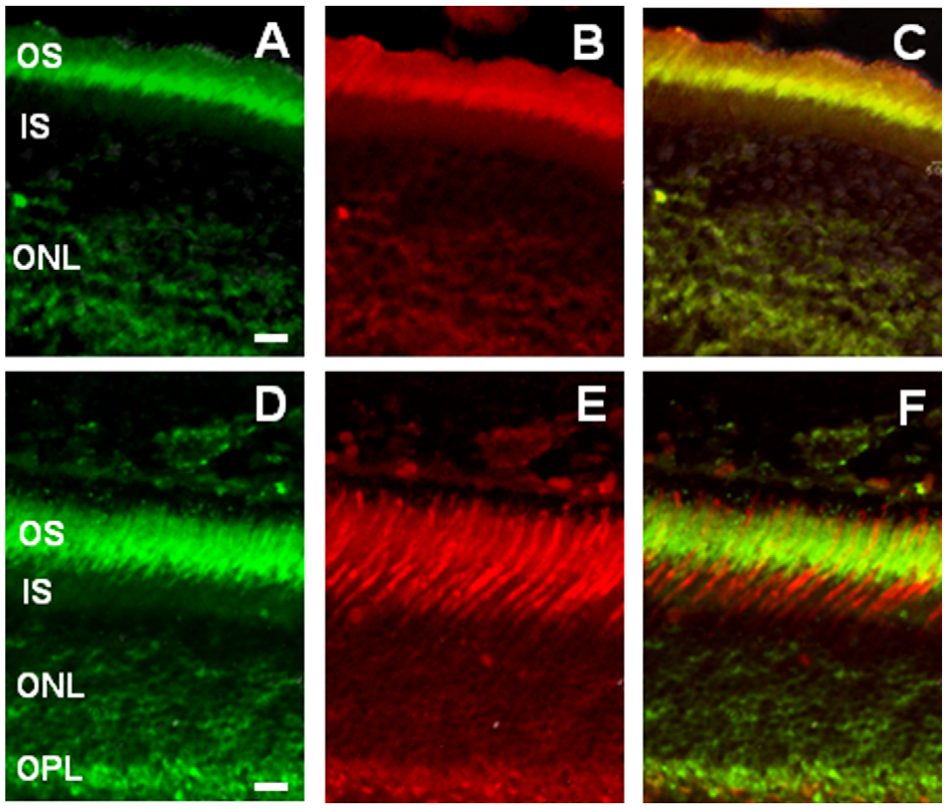
MT_1_ receptor immunoreactivity in photoreceptor cells. MT_1_ immunoreactivity was localized with rod outer-inner segments (A = MT_1_; B = rhodopsin; C = merge) and to cones outer-inner segments (D = MT_1_; E = peanut agglutinin; F = merge) and to a lesser extent, to the pedicles of the cones. The co-localization of MT1 receptor signal with rods or cones is evident in panels B and C, respectively, where the images have been merged. The bars at the bottom of the Figures represent 10 µm. Micrographs are representative of results obtained from at least three mice.

**Figure 3:**
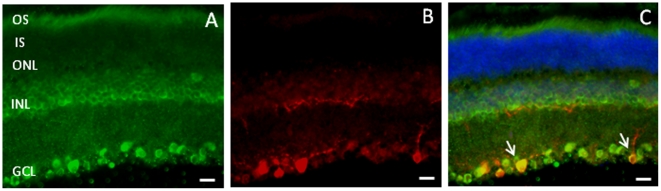
MT_1_ receptor immunoreactivity in *ip*RGCs cells. The co-localization of MT_1_ receptor with melanopsin immunoreactivity on *ip*RGCs is evident in panel C (white arrows) where the images have been merged. (A = MT_1_; B = melanopsin; C = merge). The bars at the bottom of the Figures represent 15 µm. Micrographs are representative of results obtained from at least three mice.

Western blotting of the MT_1_ receptor in the mouse retina showed an immunoreactive band of about 40 kDa in the retina of the WT, but not in the MT_1_^−/−^ mice (Figure 1, Top). The control blots, which were treated with primary antibody pre-absorbed with the antigen blocking peptide, or using retina from MT_1_^−/−^ mice did not show any band around 40 kDa. A band of approximately 80 kDa was also present in WT. (Figure 1, Top). Immunocytochemistry using the anti- MT_1_ antibody identified a strong and specific immunoreactivity in the inner/outer segment of the photoreceptors, and in the GC layer; a weak immunoreactivity was also observed in the outer plexiform layer (Figure 1A–C, Bottom). The immunoreactivity was completely abolished by pre-incubation of the antibody with the blocking peptide (Figure 1 D, E), and was absent in the retina obtained from MT_1_^−/−^ mice (Figure 1 F, G). We then investigated whether MT_1_ immunoreactivity was present on rods and cones by double labeling using a rhodopsin antibody and peanut agglutinin, a well-established marker for the cone matrix sheaths of cones. As shown in Figure 2, MT_1_ immunoreactivity was present on the rod photoreceptors (Figure 2 A–C), and on the cones (Figure 2 D–F). Finally, we investigated whether MT_1_ immunoreactivity was also present on the intrinsically photosensitive GCs (*ip*RGCs). As shown in Figure 3 A–C, we detected MT_1_ immunoreactivity on *ip*RGCs as indicated by the colocalization of MT_1_ and melanopsin immunoreactivity (Figure 3C).

**Figure 4:**
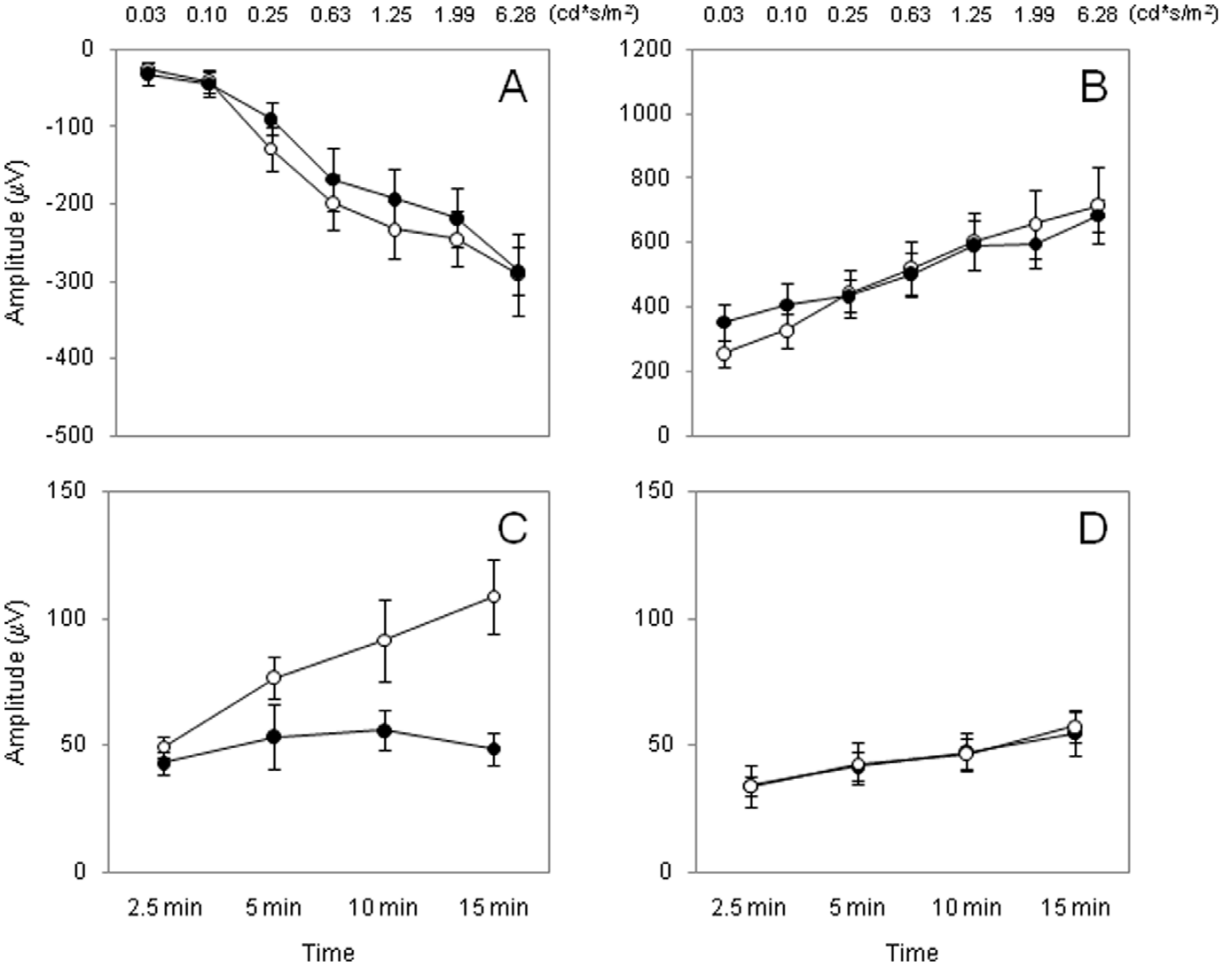
Quantification of the scotopic and photopic ERGs of WT and MT1−/− mice recorded after 18 (CT 6) and 30 (CT18) h in DD. No differences in the amplitude of the a- and b-wave were observed in the scotopic ERGs of WT mice (A, B). The photopic ERGs show a circadian regulation in WT (C, Two-way ANOVA, P<0.01, N = 6–8 for each time-point) but not in MT_1_^−/−^ mice (D, Two-way ANOVA, P>0.1, N = 6–8 for each time-point). White Circles = CT6; Black circles = CT 18.

*MT1 receptors control the circadian rhythm of the photic ERG*. WT mice did not show any circadian regulation in the scotopic ERGs (Figure 4 A–B; Two-Way ANOVA, P>0.1), whereas the photopic ERG in WT mice showed a clear circadian regulation (Figure 4 C, Two-Way ANOVA, P<0.01). The circadian regulation of the photopic ERG was absent in MT_1_^−/−^ mice (Figure 4 D, Two-Way ANOVA, P>0.1).

### Effect of MT_1_ removal on the retinal dopaminergic system

**Figure 5:**
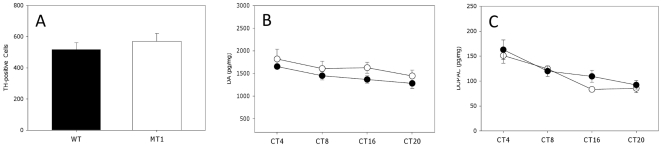
Number of TH-positive cells in the retina of WT and MT_1_^−/−^ (A) and DA and DOPAC levels measured in WT and MT_1_^−/−^ in DD. A) The number of TH-positive neurons in the retina of WT was not different from the number observed in MT_1_^−/−^ (t-test, P>0.1). The level of DA (B) and DOPAC (C) decreased as expected from day to night in both genotypes (WT: white circles; MT1^−/−^: black circles), and no differences were observed in mean levels of DA and DOPAC at each time-point between the WT and MT_1_^−/−^(Two-way ANOVA, P<0.05 in both cases). N = 9–12 for each point and genotypes.

The effect of MT_1_ removal on the dopaminergic system was determined by counting the number of TH-positive neurons, and by investigating the circadian regulation of DA and DOPAC levels. As reported in Figure 5A, the number of TH-positive neurons in the retina of WT mice was not different from the number observed in MT_1_^−/−^ (WT = 559.3+/−44.3; MT_1_^−/−^569+/−50.4, N = 3 for each genotype; t-test, P>0.1; Figure 4A). The level of DA and DOPAC were circadian in WT and MT_1_^−/−^ mice (Two-way ANOVA, P<0.05 Figure 5 B–C;), and no differences were observed in the levels of DA and DOPAC between the WT and MT_1_^−/−^ (Tukey Tests, P>0.05).

## Discussion

The focus of this study was to investigate the distribution of MT_1_ receptors in the retina using a newly developed antibody, and to determine the effects of MT_1_ signaling removal on the circadian regulation of the mouse ERGs and on the retinal dopaminergic system. Our data indicated that the newly developed MT_1_ receptor antibody detected in mouse an immunoreactive band of about 40 kDa in the retina of the WT, but not in the MT_1_^−/−^, or in control blots incubated with primary antibody pre-absorbed with the antigen blocking peptide (Figure 1). The data obtained are consistent with previously published reports in chicken, rat, and human, which indicated that MT_1_ receptor has a molecular weight around 40 kDa [14]–[16]. The fact that a band around 80 kDa was detected in the retinal extract of WT mice is intriguing and deserves some discussion. Previous studies have reported that melatonin receptors have the potential to form heteromeric complexes and these receptors were indeed among the first G protein-coupled receptors that have been shown to homo- and heteromerize in a constitutive manner when transfected in HEK 293 cell at physiological levels [2]. Therefore, it is possible that the band observed at 80 kDa may represent the MT_1_ receptor in the homodimeric or heteromeric form. However, it important to mention that a very weak band of approximately 80 kDa was also present in the blot treated with the blocking peptide and in the blot of retinal extract from MT_1_^−/−^ mice. Therefore it is also possible that this band may be the result of non-specific binding of the MT_1_ receptor antibody. Additional studies will be required to resolve this important issue.

The data obtained with immunocytochemistry (ICCH) are also consistent with previously published data using *in situ* hybridization in the mouse [4] or with ICCH in rat and human [2], [3], [15], which showed that MT_1_ receptors are widely distributed within the retina. Our study expands on these previous reports by showing that MT_1_ receptors are located on the rod, and possibly the cone photoreceptors (Figure 2). The fact that MT_1_ receptors are present on the photoreceptors further supports our previous study where we reported that MT_1_ signaling is an important modulator of photoreceptor functions and viability [4]. Previous studies have also shown that melatonin is synthesized in the photoreceptors [17]–[18], and its synthesis is directly controlled by the circadian clock [18]–[19]. The fact that MT_1_ receptors are expressed on the cells responsible for its synthesis suggests that melatonin plays an autocrine role within the photoreceptors, and on the circadian clock located in these cells. A previously published study reported that *ip*RGCs express dopamine receptors [20], and our new data expand this previous finding by demonstrating that MT_1_ receptors are also present on the *ip*RGCs (Figure 3). Such a result would suggest that the melatonin-dopamine feedback loop [21] which regulates the rods and cones circadian physiology may be also involved in the modulation of circadian rhythms in the *ip*RGCs.

Our previous study had shown that melatonin via MT_1_ actually controls the rhythms of the scotopic and photopic ERGs in C3H mice, when the mice are kept in a 12 h light: 12 h dark cycle [4], and other investigations performed with melatonin-deficient mice have shown that the amplitude of the scotopic ERGs is not rhythmic in mice housed in constant conditions (i.e., constant darkness), and thus demonstrating that the scotopic ERGs are not under circadian control [8], [9]. Since melatonin is believed to be a key regulator of circadian functions within the retina [21], we decided to investigate whether the scotopic ERG was under circadian regulation in a melatonin-proficient mouse. As shown in Figure 4 A–B the scotopic ERGs did not show any circadian regulation in our mice. On the other hand, a circadian rhythm in the photopic ERGs was observed in WT mice [Figure 4C], and was also observed in melatonin-deficient mice [8], [9] suggesting that melatonin may not be directly involved in the regulation of the photopic ERGs. Surprisingly, we observed that circadian regulation in the photopic ERG was absent in MT1^−/−^ mice (Figure 3D), indicating that in melatonin-proficient mice MT_1_ receptor signaling is necessary for the circadian regulation of the ERGs. A possible explanation for this unexpected result may be found in the possibility that elimination of the MT_1_ receptor affects the circadian regulation in the retinal dopaminergic system. As shown in Figure 5 A–C, removal of the MT_1_ receptors did not affect the number of dopaminergic neurons, nor did it affect the levels of DA and DOPAC or the circadian regulation of DA and DOPAC. Therefore, the loss of circadian regulation in the photopic ERGs observed in MT_1_^−/−^ mice is not a consequence of the loss in the circadian regulation of DA and DOPAC. Although these results may appear surprising, they are not completely unexpected since circadian regulation of DA and DOPAC is abolished in melatonin-deficient mice [11], [12], and therefore it is unlikely that the circadian regulation of the photopic ERGs in melatonin-deficient mice is driven by the circadian regulation of the retinal dopaminergic system. However, it also possible that an unknown factor, rather than melatonin, may be responsible for such a result. Future studies using MT_1_^−/−^ in melatonin deficient background may help to explain these apparently inconsistent results.

A previous investigation has shown that removal of melanopsin may affect the circadian regulation of the photic ERG [22]. Surprisingly, we observed that removal of the MT_1_ receptors produce similar results on the circadian regulation of the photic ERG, thus suggesting that melatonin may affect the circadian regulation of the photic ERG by acting on MT_1_ receptors present on the *ip*RGCs.

In conclusion our studies show that MT_1_ receptors are widely expressed in the mouse retina, and are present on the classical photoreceptors (i.e., the rods and cones) and on *ip*RGCs. Furthermore, it appears that the presence of MT_1_ receptors is required for the circadian regulation of the photic ERGs and the circadian regulation of the retinal dopaminergic system is not affected by the absence of MT_1_ receptors. Our data also suggest that MT_1_ may modulate *ip*RGCs activity.

## Materials and Methods

### Animals

C3H MT_1_^−/−^ knock-out mice homozygous for the *rd1* mutation [23], generously donated by Drs. Reppert and Weaver (University of Massachusetts Medical School), were back-crossed with C3H/f^+/+^ (WT) mice in which the *rd1* mutation was removed to produce C3H/f^+/+^MT_1_^−/−^ (MT_1_^−\−^) as described in Baba et al., [4]. The genotypes were determined according to the protocols described [23]. Mice were maintained in 12 h Light: 12 h Dark (LD) conditions (light on at 06:00 am and off at 06:00 pm) with food and water *ad libitum*. All experiments conformed to the NIH Guide on the Care and Use of Laboratory Animals, and were approved by the Institutional Animal Care and Use Committees of Morehouse School of Medicine (Protocol number 10-07).

### Tissue preparation

3–4 month old mice were sacrificed by cervical dislocation. For Immunofluorescent labeling of proteins in the retina, enucleated whole eyes of WT and MT_1_^−/−^ mice (N = 4), were fixed overnight in 4% paraformaldehyde solution in PBS, cryoprotected in 30% sucrose solution in PBS for 48 h, and embedded in Tissue Tek (OCT Compound). For western blots, the retina was removed from the enucleated eyes of each genotype (N = 3) through an incision perpendicular to the anterior posterior axis of the eye and was snap frozen in liquid nitrogen and stored at −80°C.

### Characterization of MT_1_ receptor antibody

The novel polyclonal MT_1_ receptor antibody used in this study was designed to target the C-terminal of the mouse MT_1_ (C-LQVRRRVKPDNKPKLKPQD: 218–236, GenBank accession number NC_000074), and was raised in rabbits (Pacific Immunology Corp, Ramona, CA). The specificity of the antibody was assessed by immunocytochemistry and (ICCH) and Western Blotting (WB) techniques in WT and MT_1_^−/−^. The antibody was characterized with the following experiments.

### Immunocytochemistry

All buffers used were based on PBS (0.1 M; pH 7.4). 10 µm frozen sagittal retinal sections mounted on gelatin-coated glass slides were washed in PBS (3×; 10 min each). Permeabilization of tissues was performed with 0.2% Triton X-100 in blocking buffer containing 10% goat serum for 2 h at room temperature (RT). Sections were incubated overnight at 4°C in serial dilutions (1∶50–1∶500) of the primary MT_1_ antibody in 1% blocking buffer. Sequential washing in PBS (3×) was done prior to and after incubation in secondary antibody solution (1∶500; anti-Rabbit IgG raised in Goat-FITC conjugate, Alpha Diagnostics; San Antonio TX) for 2.5 h at RT. Sections were mounted in Vectashield Mounting medium with DAPI (Vector Laboratories, Inc Burlingame CA), and imaged using an Olympus laser-scanning confocal microscope system (IX71; Olympus, Tokyo, Japan). The specificity of MT_1_ receptor antibody was checked by immunostaining retinal sections of WT mice with the MT_1_ receptor antibody pre-absorbed with its respective blocking peptide. Retinal sections of MT_1_^−/−^ mice were checked for absence of MT_1_ labeling, as a part of the validation process of the antibody.

To investigate whether expression of MT_1_ receptor is restricted to either rods, cones, or both types of photoreceptors, retinal sections of WT mice were double-labeled with MT_1_ receptor (1∶500)/Rhodopsin (1∶200; rho-4D2 mouse monoclonal antibody generated by Dr R. S. Molday, University of British Columbia, Vancouver, Canada), MT_1_ receptor (1∶500)/Rhodamine peanut agglutinin (1∶500; Vector Laboratories, Burlingame). The protocol for immunolabeling with each antibody remained the same as described above. Sections were incubated with Texas Red-conjugated goat anti-mouse IgG (1∶500; Invitrogen Carlsbad CA) and anti-Rabbit IgG raised in Goat-FITC conjugate, for detection of rhodopsin and MT_1_ receptor respectively. Immunofluorescent staining was examined under immunofluorescent confocal laser scanning microscopy (IX71; Olympus, Tokyo, Japan). Positive Immunofluorescence was detected in green (FITC) or red (Texas Red, Rhodamine). Yellow color represented colocalization of the two antigens. Photomicrographs were prepared using Olympus Fluoview Fv300 (version 4.3). Colocalization of MT_1_ immunoreactivity with *ip*RGCs was performed using a well characterized melanopsin antibody [24] kindly donated by Dr. I. Provencio (University of Virginia, Charlottesville, VA) and with FITC conjugated MT_1_ primary antibody (1∶50).

### Western Blot

All reagents for western blot analysis were purchased from Bio-Rad Laboratories (Hercules, CA, USA) unless mentioned otherwise. Six retinas of each genotype (WT and MT_1_^−/−^respectively) were used for extraction of retinal protein in 250 µl of lysis buffer (0.15 M NaCl, 2 mM EDTA, 0.15% Triton X-100 and protease inhibitor cocktail). Protein concentration was measured by Bradford assay [25]. 30 µg of linearized retinal proteins were separated by electrophoresis on a 12% Tris-HCl precast mini gel (Bio-Rad Laboratories, Hercules, CA) under reduced conditions and was subsequently electro-transferred (200 mA; 1 h) onto nitrocellulose membranes (0.8 µm pore size; Bio-Rad Laboratories; Hercules CA). The membrane was blocked in 5% skim milk solution in Tris buffered saline (TBS) containing 0.1% Tween-20 (TBST) for 2 h at RT followed by overnight incubation at 4°C in anti-MT_1_ antibody solution (1∶500 in blocking buffer). The membrane thoroughly rinsed in TBST buffer was now incubated (2 h at RT) in HRP-conjugated goat anti-rabbit IgG solution in TBST (1∶5000), processed for chemiluminescent reaction (3 min in ECL), then developed on a film. Specificity of the antibody was checked by relabeling the stripped membrane (using Restore™ stripping buffer; Thermo Scientific, Rockford IL) with MT_1_ receptor antibody pre-absorbed with its specific peptide. α-tubulin was used as internal/loading control.

### Electroretinography

Mice were maintained in constant darkness (DD) for 18 and 30 h, and then were anesthetized with ketamine (80 mg/kg) and xylazine (16 mg/kg). The pupils were dilated with 1% atropine and 2.5% phenylephrine (Sigma, St. Louis, MO, USA), and mice were placed on a regulated heating pad set at 37°C with feedback from the rectal temperature probe. The eye was lubricated with saline solution, and a contact lens type electrode (LKC Technologies model: N1530NNC) was topically applied on the cornea. A needle reference was inserted in other side of cheek, and the ground needle was inserted into the base of tail. All preparation of ERG recordings was conducted under red dim light (<3 lux, 15W Kodak safe lamp filter 1A, Eastman Kodak, Rochester, NY, USA).

All electrodes were connected to a Universal DC Amplifier (LKC Technologies model UBA-4200) and bands were filtered from 0.3 to 500 Hz. Data were recorded and analyzed by EM for Windows (ver. 8.2.1, LKC Technologies). Core body temperature was maintained in 37°C by a feedback temperature control system (FHC inc., Bowdoin, ME) during whole ERG recording. In the dark-adapted ERG protocol, seven series of flash intensity between from 0.03 to 6.28 cd*s/m^2^ were presented to the mouse eye. Flashes were generated by 530-nm green LEDs in a Ganzfeld illuminator (LKC Technologies), and intervals of flashes increased from 0.612 to 30 s as intensity of the flashes increased. Responses of 3–10 flashes were averaged to generate a waveform for each step of light intensity, and a-wave and b-wave of ERG measurement were analyzed from the trace of wave forms.

To measure the photopic ERG mice were placed in a Ganzfeld illuminator and cone-associated activity was isolated by saturating rods with 63 cd*s/m^2^ of white background light. The four series of consecutive 10 white flashes (79.06 cd*s/m^2^) were introduced at 2.5 min, 5 min, 10 min, and 15 min during the background light exposure. Background light was left on for 15 min while photopic ERGs records were measured [4]. The traces of the ERG were averaged and stored on a computer for later analysis. The amplitude of the b-wave was measured from the trough of the a-wave to the peak of the b-wave or, if no a-wave was present, from the baseline to the b-wave peak. The spectral composition and irradiance of the light was monitored by a radio-spectrophotometer (USB 2000, Ocean Optics, Dunedin, FL).

### Dopamine and 3,4-dihydroxyphenylacetic acid (DOPAC) analysis by high-performance liquid chromatography with colometric detection

Mice were maintained in a 12-h light/12-h dark cycle, with lights on from zeitgeber time (ZT) 0 to ZT 12, then maintained in total darkness (24 h per day) for 2–3 days before dissection. Animals were euthanized by cervical dislocation and the eyeballs were explanted. Then a small cut (1–2 mm) was performed at the level of the ora serrata, the lens was removed and the retina was obtained by gently squeezing the eyeball. The retinas were immediately frozen and then stored at −80°C. All manipulations on mice and tissues under conditions of darkness were performed under dim red light (15W Kodak safe lamp filter 1A). Levels of dopamine and DOPAC in mouse retina were determined by ion-pair reversed-phase high-performance liquid chromatography (HPLC) with colometric detection (guard cell set at 0.6 V, and colometric analytical cell set at 0.3 V) as described by Pozdeyev et al. [13]. Retinas were homogenized in 100 µl of 0.2 N HClO_4_ containing 0.01% of sodium metabisulfite and 25 ng/ml of internal standard 3,4-dihydroxybenzylamine hydrobromide. After centrifugation at 15,000× g for 10 min, an 80 µl aliquot of supernatant was analyzed. The separation was performed on an Ultrasphere ODS 250×4.6 mm column, 5 µm (Beckman Coulter, Fullerton, CA, USA) with a mobile phase containing 0.1 M sodium phosphate, 0.1 mM EDTA, 0.35 mM sodium octyl sulfate, 5.5% acetonitrile (vol/vol), pH 2.7. External standards of dopamine and DOPAC were analyzed in each experiment.

Finally, to determine whether removal of MT_1_ receptors affects the number of dopaminergic neurons in the retinas from WT and MT_1_^−/−^ mice, retinal samples from both strains were stained as whole mounts with a tyrosine hydroxylase (TH) antibody (Chemicon, Temecula, CA). TH-positive neurons were revealed with an Alexa 568-conjugated secondary antibody and imaged with a Leica SP2 confocal microscope using a 20× objective (HC PL APO 20×/0.70). Montages of tile images covering the whole retinal surface were obtained acquiring TH-positive cell stacks of 10 microns and collapsing in a maximal projection. TH-positive neurons were counted by means of a Metamorph image analysis routine. Statistical analysis was performed using one-way ANOVA (p<0.05 and p<0.01).
